# Developmental changes and novelties in ceratophryid frogs

**DOI:** 10.1186/s13227-016-0043-9

**Published:** 2016-02-27

**Authors:** Marissa Fabrezi, Silvia Inés Quinzio, Javier Goldberg, Julio César Cruz, Mariana Chuliver Pereyra, Richard J. Wassersug

**Affiliations:** Instituto de Bio y Geociencias (IBIGEO), Centro Científico Tecnológico CONICET-Salta, 9 de Julio 14, 4405 Rosario de Lerma, Salta Republic of Argentina; Department of Medical Neuroscience, Dalhousie University, Halifax, NS Canada

**Keywords:** Growth, Development, Morphological novelty, Metamorphosis, Anurans

## Abstract

The Neotropical frog genera *Ceratophrys*, *Chacophrys* and *Lepidobatrachus* form the monophyletic family Ceratophryidae. Although in- and out-group relationships are not fully resolved, the monophyly of the three genera is well supported by both morphological and molecular data. Much is known about the morphology of the ceratophryids, but there is little comparative information on how modification of a common ancestral developmental pathway played a role in shaping their particular body plans. Herein, we review morphological variation during ceratophryid ontogeny in order to explore the role of development in their evolution. The ceratophryids are collectively characterized by rapid larval development with respect to other anurans, yet the three genera differ in their postmetamorphic growth rates to sexual maturity. Derived traits in the group can be divided into many homoplastic features that evolved in parallel with those of anurans with fossorial/burrowing behaviors in semiarid environments, and apomorphies. Morphological novelties have evolved in their feeding mechanism, which makes them capable of feeding on exceptional large prey. *Lepidobatrachus* is unusual in having reduced the ecomorphological differences between its larvae and adults. As a result, both the larvae and the frog are similarly able to capture large prey underwater. Some unique features in *Lepidobatrachus* are differentiated in the tadpole and then exaggerated in the adult (e.g., the posterior displaced jaw articulation) in a manner unobserved in any other anurans.

## Background

Based on morphological and molecular data, the South American anuran genera *Chacophrys* Reig and Limeses 1963 (one species), *Ceratophrys* Wied-Neuwied 1824 (eight species) and *Lepidobatrachus* Budgett 1899 (three species) constitute a monophyletic clade, the Ceratophryidae. *Ceratophrys* species are distributed in tropical areas with *Ceratophrys cranwelli* living with *Lepidobatrachus* spp. and *Chacophrys pierottii* in the semiarid lowlands of the Chaco region.

The monophyly of the group, often referred to as horned frogs, was proposed by early researchers [1–5] and ratified by more recent cladistic analyses [6–10]. However, two controversies remain regarding the relationships of the group: (1) the relationships between the three genera and (2) the group’s relationship with other anurans.

**Fig. 1:**
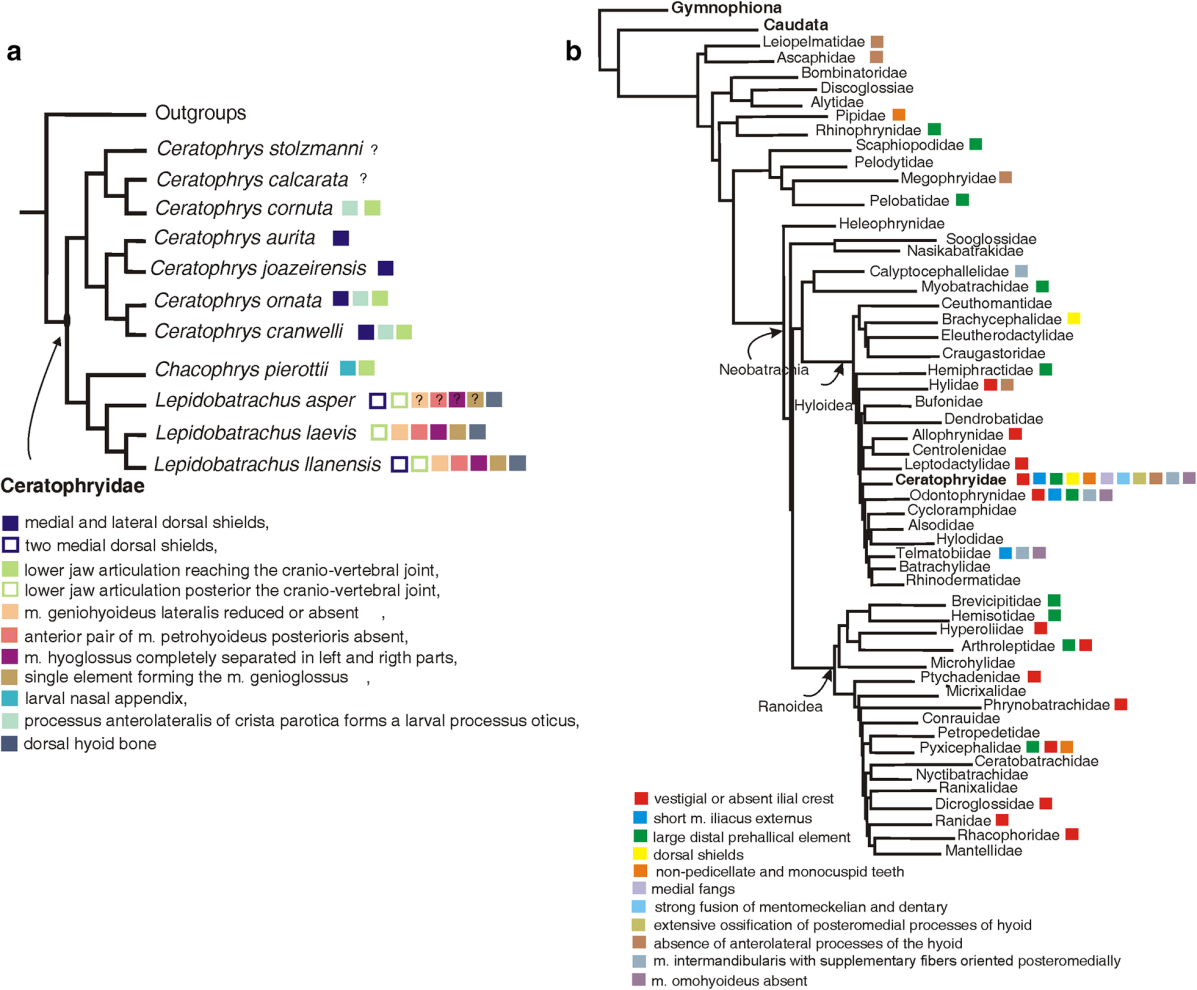
Recent molecular phylogenies for **a** the Ceratophryidae and **b** the Anura. Selected traits that are proposed as synapomorphies for the certophryids (discussed in the text) are mapped onto the two cladograms. The well-supported monophyly for the three genera in the Ceratophryidae (**a**) and the relationships of the 12 extant species within the family permit interpretation of the changes in development that lead to morphological diversity in the family (although questions remain about the relationship of *C. pierottii*) [10]. Within the Anura (**b**), the Ceratophryidae appear to hold a relatively basal position among South American hyloid clades [9]. Despite many diagnostic apomorphies in the Ceratophryidae, the homoplastic and autapomorphic traits make it difficult to pinpoint the origin of these frogs. The hypothesized relationships in these figures represent the phylogenetic framework for the morphological comparisons presented in this review

Studies of the Ceratophryidae have alternatively proposed the basal taxon to be *Ceratophrys* [5, 7, 9–11], *Chacophrys* [11] or *Lepidobatrachus* [1, 4, 8]. More recently, molecular data of the 12 extant species were reanalyzed within a large taxon sample, and the monophyly of *Ceratophrys* and *Lepidobatrachus* (Fig. 1) was corroborated [10]. In this phylogeny, the monotypic *Chacophrys* sits as the sister taxon of *Lepidobatrachus*, but with Jackknife frequency <50 % (Fig. 1a).

When the relationship of the Ceratophryidae to other anurans has been examined, the South American horned frogs have been variously proposed as: a basal taxon within Bufonidae [1]; related to Leptodactylidae [2, 4, 5] or to certain hylids, but with only weak support [6]; a basal group of Neobatrachia [7]; the sister group of *Odontophrynus* [4]; the sister group of Batrachyilinae [8]; the sister group of Telmatobiinae [12]; a sister lineage of a large clade within Hyloides [9] (Fig. 1b); and even a basal group of Hyloides [10].

Two Cretaceous fossils have been attributed to the Ceratophryidae and are the oldest fossils associated with the family. These are *Beelzebufo ampinga* from the Upper Cretaceous (Maastrichtian) Maevarano Formation of Madagascar [13, 14] and *Baurubatrachus pricei* from the Upper Cretaceous of Brazil [15]. Other more recent fossils have been placed within the Ceratophryidae. Specimen assigned to *Wawelia geroldhi*, from Miocene sediments of northern Patagonia in Argentina [16], seems to represent a juvenile anuran with some features like extant ceratophryids. Other late Miocene specimens have been attributed to *Ceratophrys* [17–20] and *Lepidobatrachus* [21, 22]. Those specimens, plus independent molecular data [5, 23], indicated that both genera were well differentiated by the Miocene.

**Fig. 2:**
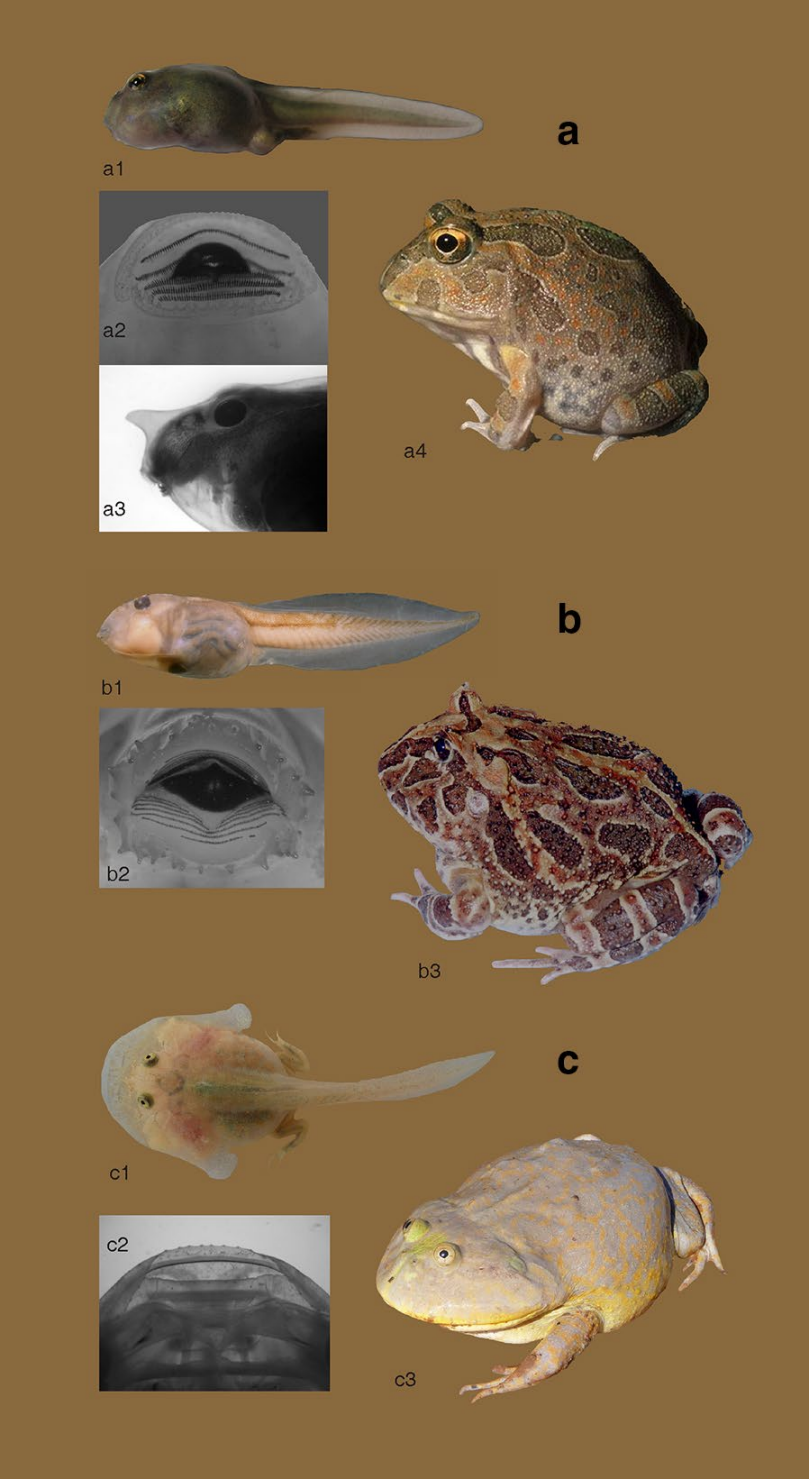
Morphological variation among larval and adult ceratophryids. Figures are not in scale. **a** *Chacophrys pierottii.* *a1* The *C. pierottii* larvae resemble a typical type IV tadpole [26]. *a2* *C. pierottii* oral disk. The oral disk bears a single and continuous row of marginal papillae. The labial tooth row formula is 1 (1 + 1)/(1 + 1) 2. *a3* Lateral view of *C. pierottii* larval head. An unusual and variable feature in *C. pierottii* larvae is a cutaneous nasal appendix of unknown function that projects forward between the nostrils in some individuals [27]. *a4* Adult *C. pierottii.* Frogs of this species reach snout-vent lengths of about 55 mm. **b** *Ceratophrys cranwelli*. *b1* The *Ceratophrys* tadpole has most of the features of type IV tadpoles, but the larva is modified for a macrophagous life style. *Ceratophrys* tadpoles first bite small prey and then ingest them whole, or chew larger prey into pieces before ingestion [28]. In *Ce. ornata* and *Ce. cranwelli*, the tadpoles emit underwater sounds that are thought to be a mechanism for avoiding cannibalism [29, 30]. *b2* *Ce. cranwelli* oral disk. The oral disk has a single row of marginal papillae, which are few and spur-like. The labial tooth row formula is 3 (3 + 3)/(4 + 4) 3. The keratinized jaw sheaths are serrated. *b3* Adult *Ce. cranwelli*. The mature frog is large (snout-vent length up to 130 mm), stout and aggressive. **c** *Lepidobatrachus laevis.* c1. The *Lepidobatrachus* tadpole has a flattened head and an extremely wide mouth, such that the maximum width of the head is at the level of its lower jaw articulation. The feeding mechanism consists of swallowing prey whole [31]. The tadpole’s branchial chambers open in bilateral cutaneous lateral flaps in which the forelimbs develop. Fast tail movements allow for rapid escape from predators (without the sound emissions seen in *Ceratophrys*). Cannibalism, as a strategy to survive when heterospecific prey are limited, has been witnesses in tadpoles of *L. llanensis* [28]. *c2* *L. laevis* oral disk. The supralabial and lower jaw cartilages of the larva are transversally elongated. There is a single row of marginal papillae, which are small and few. The keratodonts are absent, and there is a vestigial, serrated keratinized upper jaw sheath. *c3* Adult *L. laevis.* The mature frog, like the larvae, is dorsoventrally flattened with its eyes and nostrils positioned dorsally. Females *L. laevis* may reach snout-vent length of ~120 mm

The adults of extant ceratophryids are characterized by medium to large body size (Fig. 2). The three genera share as well several derived morphological features associated with a terrestrial and fossorial life, plus adaptations for feeding on large prey [7, 10, 24, 25] (Fig. 1). The tadpoles of these genera are, however, remarkably distinct (Fig. 2). *Ceratophrys* spp. have macrophagous and specialized carnivorous larvae with robust keratinized mouthparts [32, 33]. *Chacophrys* has a more typical, generalized, suspension feeding tadpole [27, 34], and *Lepidobatrachus* larvae are obligatorily megalophagous [33], feeding upon living nekton, including other tadpoles. *Lepidobatrachus* tadpoles display many morphological features for capturing very large prey that are exceptional among anurans [30, 32, 35, 36]. The uniqueness of *Lepidobatrachus* tadpoles resulted from evolutionary changes in several specific developmental pathways that occurred simultaneously or sequentially from a generalized larval type [36, 37].

Despite much data supporting ceratophryid monophyly, the evolution of these anurans remains enigmatic. Although much is known about their morphology, there is little comparative information on how development played a role in shaping the divergent ceratophryid body plans. What in particular has not been explored is the interplay between pre- and postmetamorphic development. Uninvestigated is how these developmental pathways have influenced each other to arrive at their variously shared and unique features of adult and larval ceratophryids.

Here, we review information on variation among ceratophryid ontogenies to address two interrelated questions: (1) How did modification of development pathways play a role in the differentiation of ceratophryid genera? and (2) How did those developmental pathways contribute to the evolutionary history that distinguishes the ceratophryid from the other hyloid lineages? We provide data to (1) illustrate how development can evolve and (2) present a case study of how the detailed knowledge of morphological variation during development strengthens evolutionary studies.

**Fig. 3:**
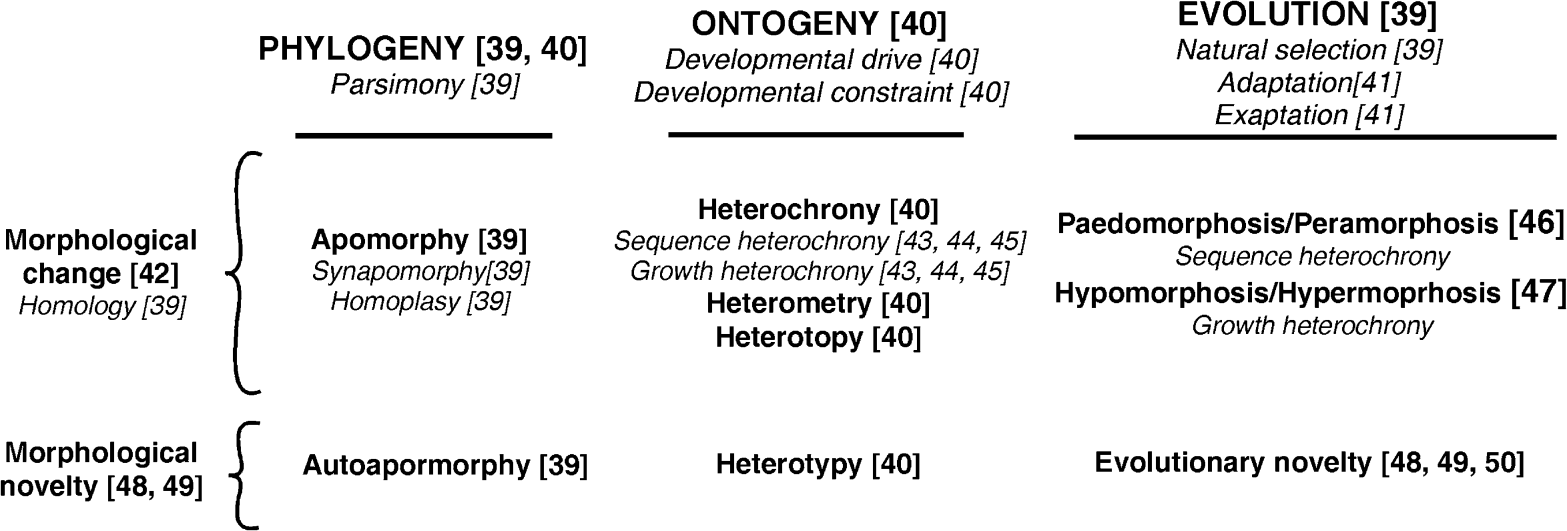
Major terms used to explain the morphological animal variation from different approaches. The scheme summarizes equivalences among these terms offering explanations and/or hypotheses to understand changes in the form through development and evolutionary time. The numbers in brackets refer to references where the terms are more extensively discussed. For brief definitions, see “Appendix”

Variation both between organisms and within organisms as they develop has provided enough information to yield a conceptual framework for understand how developmental pathways for ceratophyrids have evolved through time. General terminology used to describe the interplay between evolution and development in general is presented in Fig. 3. It should be noted that several of these terms have been used in slightly different ways by different authors. As such, we follow the definitions of these terms presented and referenced in “Appendix.”

## Developmental and growth rates

From an ontogenetic approach, heterochrony has become a focal concept that integrates many areas of evolutionary biology [45]. Different definitions, however, have been used to explain heterochrony (cf. “Appendix”), and controversies have emerged since heterochronic patterns cannot be unequivocally classified without information of the timing (age) of developmental events in the ancestral and descendant ontogenies [45].

**Fig. 4:**
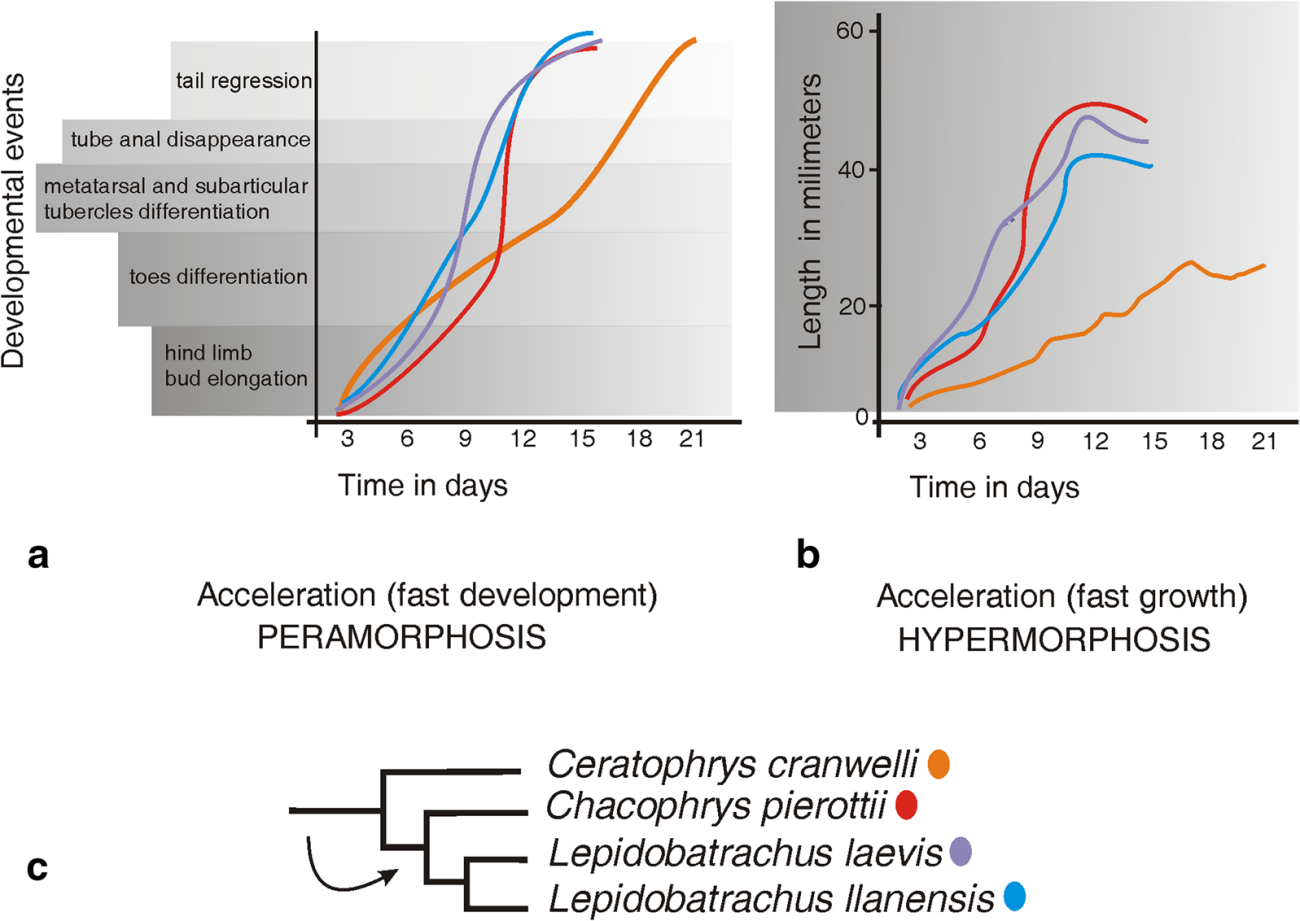
Heterochronic variation in shape and size during larval development among ceratophryids. **a** This plot depicts developmental changes versus developmental timing. The offset of larval development occurs at the moment when the tail is lost. **b** This plot, in contrast, shows size variation versus developmental timing (growth). The final larval body size is achieved at metamorphosis when the tail regresses [52, 53]. **c** Different rates determine when the final larval size and shape are achieved [11, 50]. These *curves* indicate that growth and development are accelerated in *Chacophrys* and *Lepidobatrachus* compared with the same processes in *Ceratophrys.* This perspective on ceratophryid development fits with their hypothesized phylogeny [10]

As heterochrony produces morphological changes in shape and size of a trait relative to the ancestral ontogeny, there are some useful concepts to describe heterochrony even when developmental timing is unknown [42–44]. Sequence heterochrony and growth heterochrony facilitate the distinction between variation in shape (as development) and variation in size (as growth), and both, as noted below, appear to have occurred in the evolution of the Ceratophryidae, following the terminology (Fig. 3; “Appendix”), and they are consistent with the evolutionary processes of peramorphosis and hypermorphosis [7, 11, 50] (Fig. 4).

The importance of growth heterochrony for distinguishing ceratophryids from other anurans was demonstrated in a comparison of the larvae from 20 species (five anuran families) that co-occurred with ceratophryids in the Chaco in South America. Data on size at metamorphosis and duration of the larval period for most non-ceratophryid species in this sample suggested similar growth rates [50], i.e., with development to metamorphosis taking between 20 and 75 days for 15 of those 20 species and larval body sizes varying between 9 and 25 mm. By comparison tadpoles of *Chacophrys pierotii* and *Lepidobatrachus* spp. reach metamorphosis between 15–18 days and *Ceratophrys cranwelli* in 20–24 days, with body sizes ranging from 25 to 45 mm [50] (Fig. 4).

Precise data on age at sexual maturity and postmetamorphic growth rates are not available for any ceratophryids in the wild. However, it is possible to infer the age of reproductive adults from wild-caught specimens from lines of arrested growth. Such data suggest that developmental and growth rates after metamorphosis differ greatly among ceratophryids. In *Lepidobatrachus* spp., sexually mature individuals of 5–6 years are considerably larger than sexually mature *C. pierottii* of the same age [11]. The ages for mature males of *Ce. cranwelli* vary between 11 and 14 years old with sizes slightly larger than those of *Lepidobatrachus laevis* at 6 years [11].

**Fig. 5:**
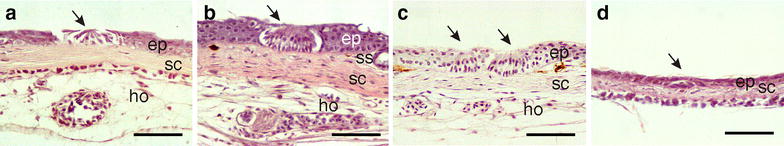
Variation in the number of layers of the dorsal integument and neuromast size from ceratophryids. Dermal histology in larvae at Gosner stage 37 for **a** *Ceratophrys cranwelli*, **b** *Lepidobatrachus laevis*, **c** *L. llanensis* and **d** *Chacophrys pierottii*. The variation in the size of neuromasts in the dorsal lateral line (*arrows*) seems to be related to integument thickness. In *Ce. cranwelli and Lepidobatrachus* spp., the epidermis is pluristratified, and the dermis has spongiosum and compactum strata in *L. laevis*. In *C. pierottii*, there are only two epidermal layers and a thin compact stratum in the dermis. *ho* hypodermis, *sc* stratum compactum, *ep* epidermis, *ss* stratum spongiosum. *Bar* equals 50 μm

In ceratophryids, accelerated differentiation and growth has also been described for many organ systems [11, 51]. An example is the early acquisition of mature skin features—i.e., three or more epidermal layers, a well-differentiated dermis, and a thick stratum compactum—in larvae of *Ce. cranwelli* and *Lepidobatrachus* spp. [11, 36] (Fig. 5). Furthermore, the size of the neuromasts appears to be related to these integumentary features, with larger organs present early in *Lepidobatrachus* spp. Conversely, small neuromasts are observed in species with typical larval skin, such as *C. pierottii* [36]. In *L. laevis*, sequence heterochrony has led to the retention of the lateral line system through metamorphosis, with the size of the neuromasts similar to that of the larval stages [36].

## Morphological evolution related to the postaxial skeleton indicative of homoplasy

In anurans, with the exception of axial musculature that changes with metamorphosis, the appendicular musculoskeletal system develops and grows independently of the larval body plan [54]. This can be understood within the context of modular organization of development [41, 55]. For developing anurans, the postaxial musculoskeletal system is divided into two separate units: (1) the trunk and tail that collectively serve for swimming and (2) the appendicular system that develops to serve adult tetrapod locomotion.

In ceratophryids, there are a few derived features in the postaxial skeleton (Fig. 1b). These include the absence of a crest on the ilium, the presence of a very short muscle iliacus externus [54, 56], a strong prehallical element for digging and the presence of dorsal shields in some species of *Ceratophrys* and *Lepidobatrachus* [10, 24, 57].

**Fig. 6:**
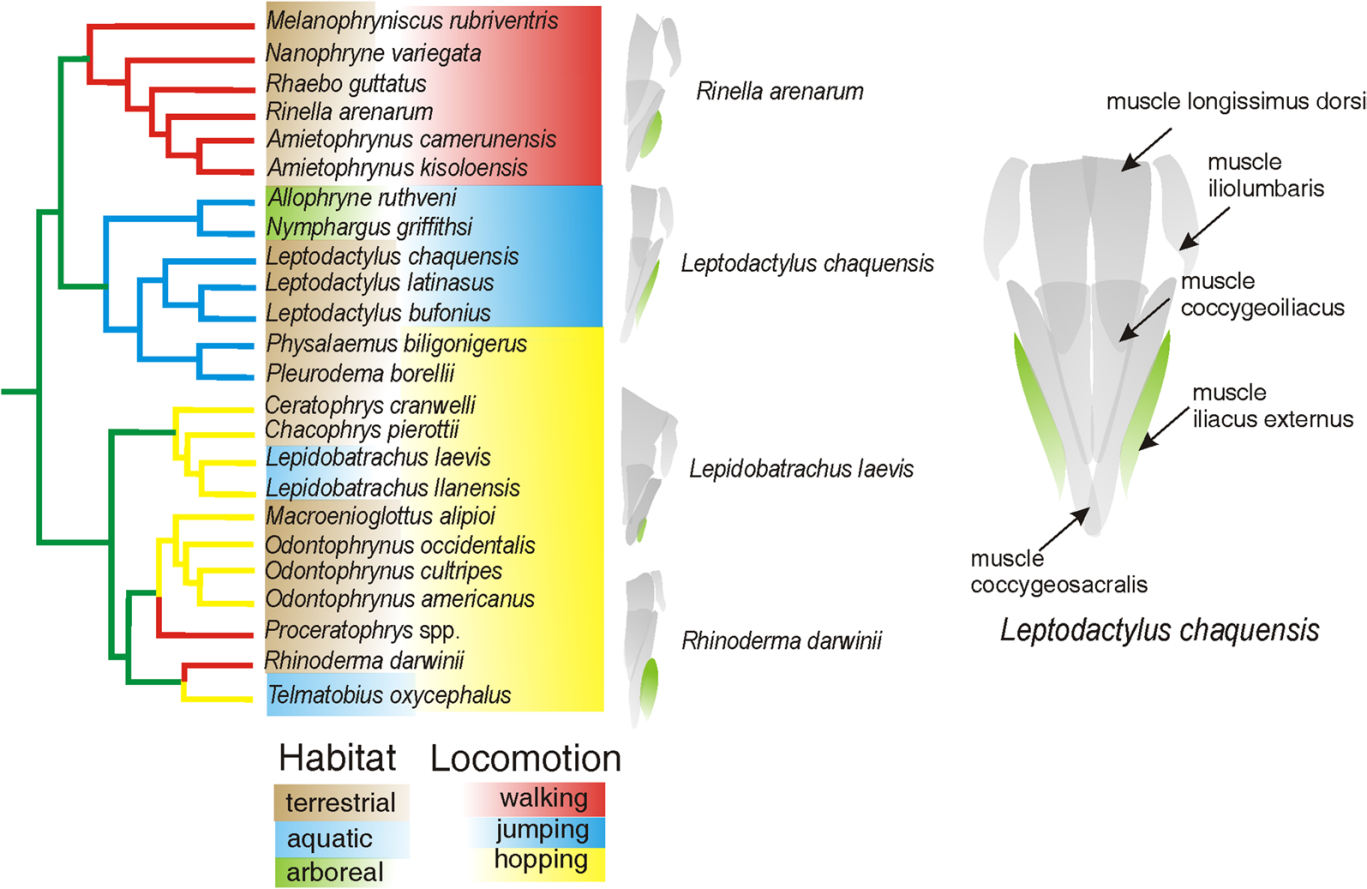
Variation in the muscle iliacus externus among selected hyloids. On the *left* is shown a phylogenetic tree for hyloid anurans [9], where *color* fields have been added to indicate the ecology and dominant locomotor patterns for the various taxa. For reference, on the *right* is shown the musculature in a representative hyloid taxon in dorsal view, with the most common muscle pattern seen in jumping frogs. *Color* branches of the tree indicate different states of a fundamental morphological character that relates to the locomotor behavior of anurans; i.e., notably the relative length of the muscle iliacus externus [54, 56, 58] which is colored in *green*. Three states are recognized for that muscle. *Blue* represents the condition in more saltatory frogs, where the origin of the muscle iliacus externus is on the anterior half of the iliac shaft and the muscle covers more than 70 % of the iliac shaft length. *Red* represents the condition in frogs that predominantly walk, where the origin of the muscle iliacus externus is on the middle of the iliac shaft and the muscle covers between 40 and 70 % of the shaft. Lastly, *yellow* represents the state seen in some hopping frogs, where the muscle iliacus externus originated on the posterior half of the iliac shaft. According to the phylogeny [9], the shortening of the muscle iliacus externus would have evolved at least three times in the clade that includes three hyloid linages: ceratophryids, *Odontophrynus* + *Macrogenioglottus*, and *Telmatobius*. This clade is formed by terrestrial (or secondarily aquatic) taxa that predominantly hop

A shortened muscle iliacus externus has evidently evolved many times within the hyloids (Fig. 6). The muscle is progressively diminished within *Lepidobatrachus* in the sequence *L. llanensis*, *L. asper* and *L. laevis* [58].

**Fig. 7:**
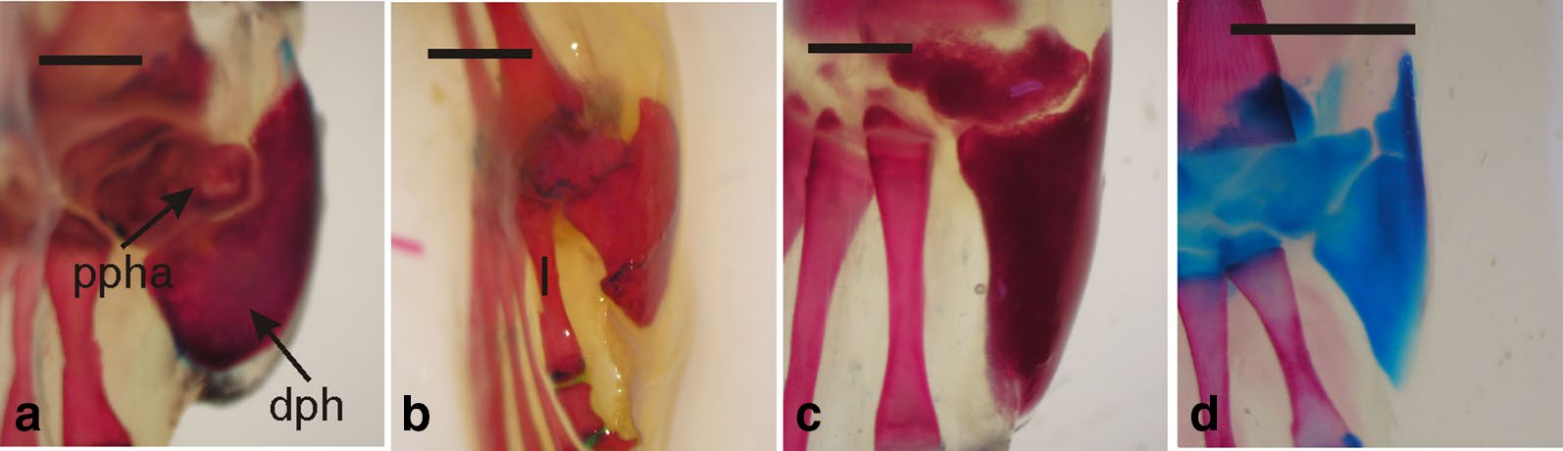
The prehallux is modified to support a keratinized spade for digging in ceratophryids. Cleared and stained specimens showing the spade on the foot in dorsal view of **a** *Ceratophrys cranwelli*, adult female; **b** *Chacophrys pierottii*, adult male; **c** *Lepidobatrachus laevis*, adult male; and **d** *Lepidobatrachus llanensis*, recently metamorphosed individual. Keratinization occurs earlier in *L. llanensis*, i.e., when digits become completely separated and the internal metatarsal tubercle is well differentiated. The same process occurs at the beginning of metamorphosis in *L. laevis* and *C. pierottii*, and after metamorphosis in *Ce. cranwelli*. *dpha* distal prehallical element, *pph* proximal prehallical element. *Bar* equals 2 mm

The prehallux is formed by two elements: The proximal one is spherical and the distal one is axehead-shaped. The distal prehallux provides support for a keratinous “spade” used for burrowing by fossorial anurans. The distal prehallux has a pronounced dorsal process that develops early and is well defined before metamorphosis (Fig. 7). In addition to the ceratophryids, a prehallux with these features occurs in species within the genera *Spea*, *Scaphiopus*, *Odontophrynus*, *Astylosternus*, *Arthroleptis*, *Hemisus*, *Scaphiophryne*, *Breviceps*, *Pyxicephalus*, *Rhinophrynus dorsalis* and *Neobatrachus pictus* [59–64]. Notably, these taxa largely occupy semiarid regions, where burrowing by the frogs into the ground is protective against desiccation during the drier times of the year.

**Fig. 8:**
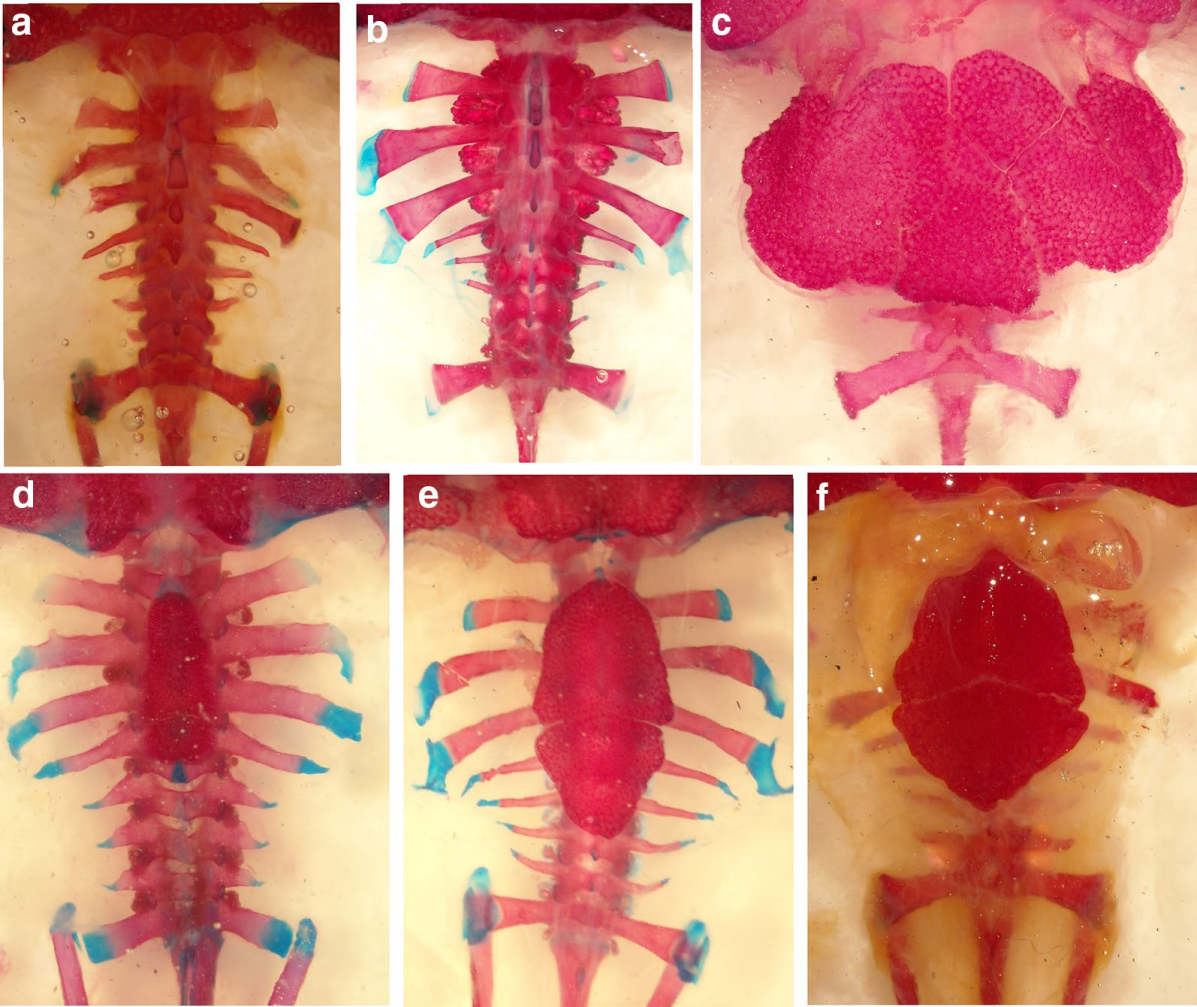
The vertebral column and overlying dorsal shields (when present) in cleared and stained ceratophryids. **a** *Chacophrys pierottii*, adult male, **b** *Lepidobatrachus laevis*, adult male, **c** *Ceratophrys cranwelli*, adult male. **d** *L. llanensis*, metamorphic individual, **e** *L. llanensis*, metamorphic juvenile and **f** *L. llanensis*, adult male. In *Chacophrys* and *Lepidobatrachus*, dorsal shields are absent and neural spines of vertebrae II–IV are flattened. *L. llanensis* bears two or three medial dorsal shields that are differentiate before metamorphosis. In *Ce. cranwelli*, the armor is composed of medial and lateral shields covering vertebrae II–VII and their transverse processes, which develop in the postmetamorphic juvenile stage

Mineralized structures in the integument, such as a calcified layer, cranial co-ossification and dorsal shields on presacral vertebrae, have similarly been associated with reducing evaporative water loss in anurans [65–71]. Dorsal shields are rare among extant anurans, but have been found in some ceratophryids (Fig. 8), some brachycephalids and few dendrobatids [7, 14, 57, 72, 73]. Dorsal shields also occur in temnospondyl amphibians of the Paleozoic [65, 74]. In ceratophryids, dorsal shields develop via intramembranous ossification and differ from the dorsal shields in *Brachycephalus ephippium* [57, 73]. In *Lepidobatrachus* spp., two or three medial shields arise during the larval stages in an antero-posterior direction from osteoblasts that in turn arise from mesenchymal cells within the hypodermis. In *Ceratophrys cranwelli*, there is a sequential addition of bony shield elements, beginning with the medial plates and progressing to the lateral and caudal ones that appear in advanced postmetamorphic stages [57]. Among Ceratophryidae, only *Ce. aurita*, *Ce. cranwelli*, *Ce. joazeirensis*, *Ce. ornata*, *L. asper* and *L. llanensis* bear dorsal shields. It has been proposed that they evolved two or more times in the history of the family [10].

These postcranial morphological traits of ceratophryids are homoplastic and, as already noted, are commonly associated with terrestrial/fossorial habits and resistance to desiccation in anurans (Fig. 1b). Consistent with that is the fact that *Chacophrys* and *Lepidobatrachus* are the only anuran genera solely endemic to the semiarid South American Chaco region [50]. Furthermore, the presence of a cocoon as a mechanism to prevent water loss during estivation, even in *Ceratophrys* spp. from humid environments, supports the idea that the Ceratophryidae originated and diversified in a semiarid environment comparable to what occurs in the contemporary Chaco [10].

## Morphological evolution related to the visceral arches and feeding indicative of synapomorphies

The analysis of the derived characters in the horned frogs (Fig. 1), both in larvae and adults, and particularly those distinct to *Lepidobatrachus*, reveals many developmental changes. In *Lepidobatrachus*, new ontogenetic trajectories are associated with a wealth of anatomical structures associated with the organisms’ pre- and postmetamorphic feeding mechanism. These changes result variously from developmental variation that is recognized as heterochrony, heterometry, heterotopy, and heterotypy or some combination of these developmental processes (Fig. 3; “Appendix”). The occurrence of heterochrony, heterotopy and heterometry may be detected by comparisons between ontogenies and/or adult traits where these processes have consequences in the final shape. For example, heterotopy and heterometry are identified in adult *Leptidobatrachus* characters in which spatial relationships (e.g., nerves in relation to muscles) are distinct, or morphometric differences appear (e.g., allometry in lower jaw length, ossification of hyoid plate). Heterochrony may occur without morphological consequence in adult traits and requires developmental sequences for interpretations (e.g., sexual maturity). In contrast, heterotypy is observed in unique traits that have their own developmental sequence. Heterotypy, as a developmental phenomenon, is a new ontogenetic trajectory and represents an autapomorphy in a monophyletic lineage.

**Fig. 9:**
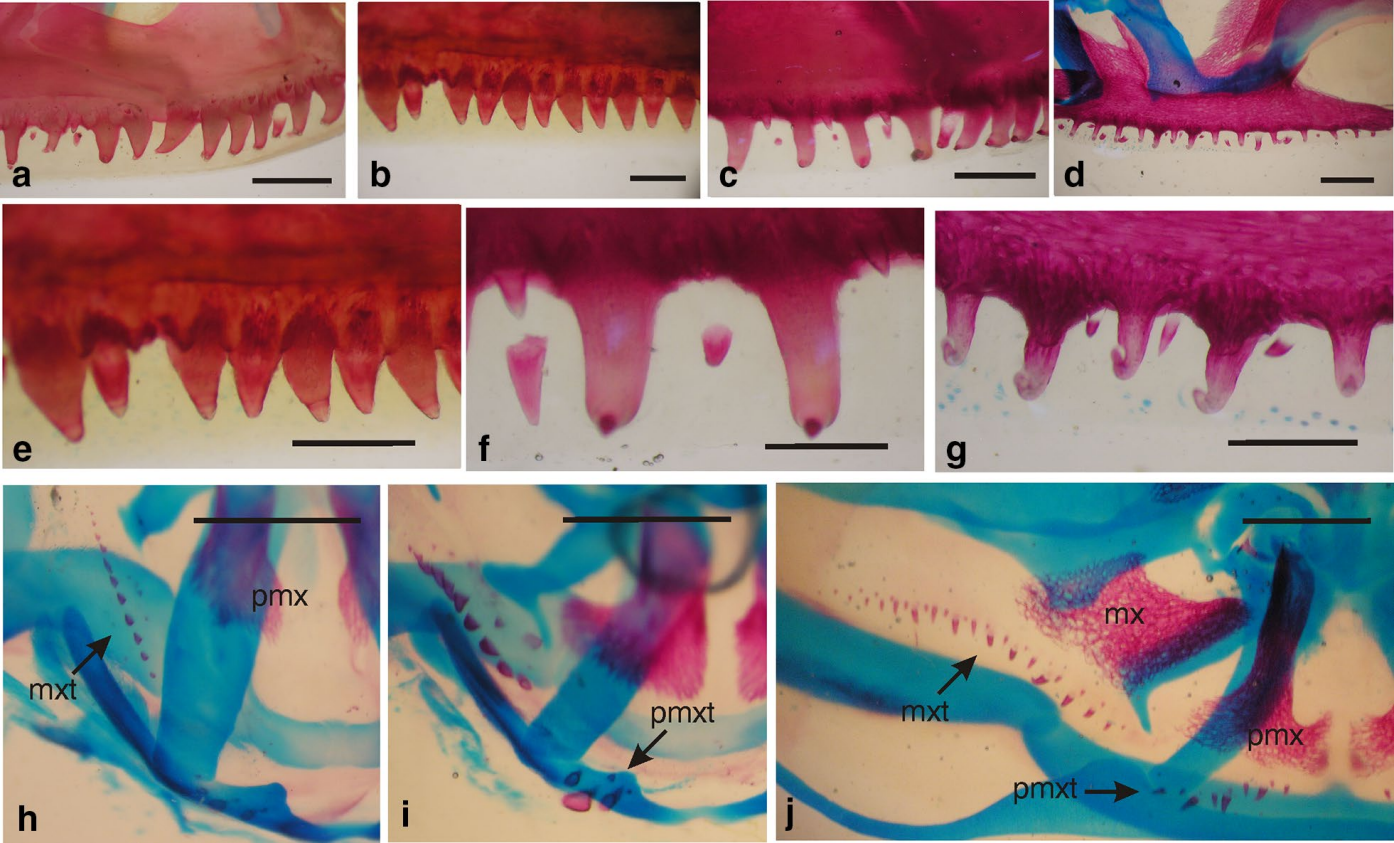
Premaxillary and maxillary teeth in ceratophryids. Whole mounts stained for cartilage and bone. **a** *Ceratophrys cranwelli*, adult female. **b**, **e** *Chacophrys pierottii*, adult male. **c**, **f** *Lepidobatrachus laevis*, adult male. **d**, **g** *L. llanensis*, recently metamorphosed individual. **h**, **i** Dorsal view of the snout of *C. pierottii* at larval Gosner Stages 39 and 41. Germs of maxillary and premaxillary teeth appear already calcified before differentiation the maxillary bone. **j** *L. laevis* tadpole before the beginning the metamorphosis with teeth germs and the incipient premaxillary and maxillary bones. In ceratophryids, the early differentiations of teeth within the larval dermis illustrate the capability of the integument to give rise to ectopic ossifications before metamorphosis. *mx* maxillary, *mxt* maxillary teeth, *pmx* premaxillary, *pmxt* premaxillary teeth. *Scale bars* in **a**–**d**, **e**–**g** equals 1 mm, **h**–**j** equal 2 mm

In ceratophryids, the upper jaw bones bear non-pedicellate, monocuspid teeth (Figs. 1b, 9) that are differentiated and calcified in late larvae stages. At metamorphosis, they immediately become attached to the premaxillary and maxillary [62, 63, 75]. Such early differentiation and rapid calcification has similarly been noted for the non-pedicellate monocuspid teeth in the hyperossified *Pyxicephalus adspersus* [76] and pipids [77]. Most anurans in contrast have pedicellate bicuspid teeth that appear at the end of metamorphosis with a persistent non-calcified zone that divides the crowns from the pedicels [78]. The shift from the generalized anuran dental morphology to the ceratophyrid pattern fits with an early onset and accelerated rate of calcification of dental germs (i.e., sequence heterochrony).

**Fig. 10:**
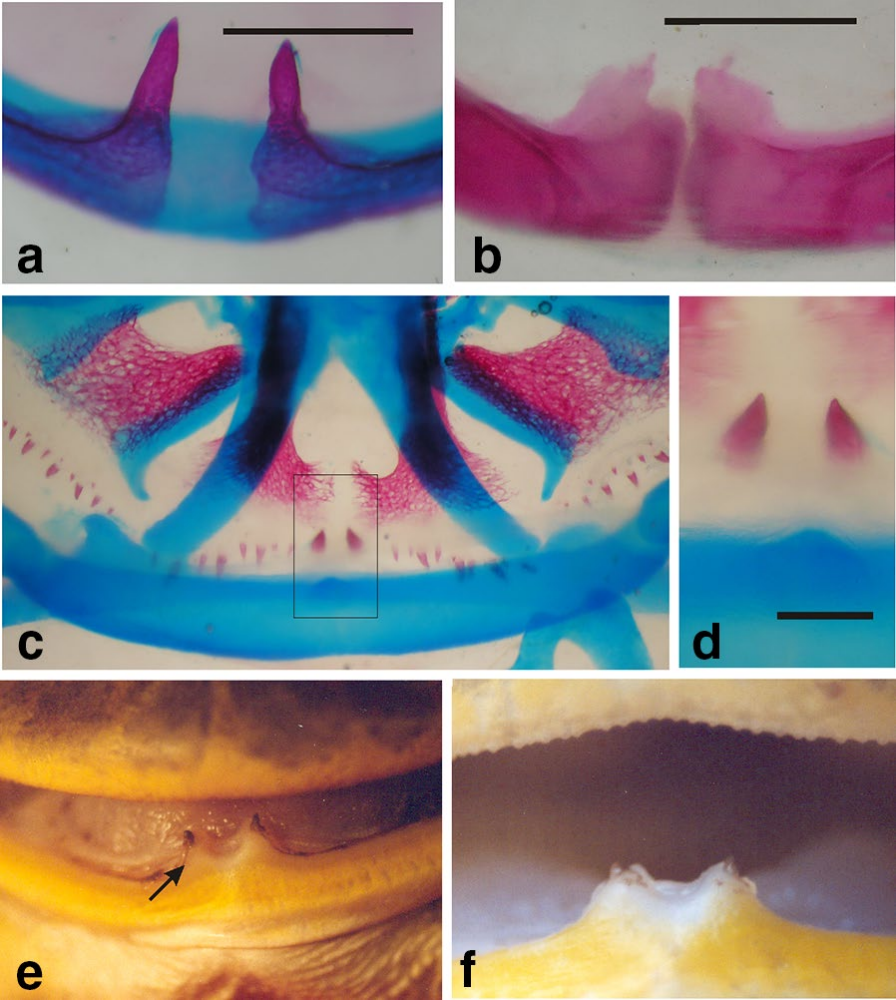
Ceratophyrid fangs in whole mounts stained for cartilage and bone, and in living specimens. **a** Lower jaw of a recent metamorphosed *Lepidobatrachus llanensis* in which the cartilaginous mandibular symphysis and pointed fangs are shown, bar is equal 1 mm. **b** Lingual view of the mandibular symphysis in *Ceratophrys cranwelli*. The fangs are stout, the mandibular bones are strongly fused, and the mentomeckelian elements are not visible; *bar* is equal 1 mm. **c** Ventral view of the lower jaw in a metamorphic specimen of *L. laevis*. The image shows the fang germs located in the lingual face of the lower jaw adjacent to the infrarostral cartilage. Calcification of the fangs precedes the calcification of lower jaw bones. **d** Detail of the fang germs in the specimen in **c**, *bar* is equal 5 mm. **e** Frontal view of the fangs with an integumentary cover in an adult specimen of *L. laevis.* **f** The fangs and the serrated teeth suggest powerful jaws to hold and subdue prey in adult of *L. laevis*. The development of fangs arising from the lower jaw in these frogs necessitated changes in the premaxillary and maxillary bones. When the mouth is closed, the fangs rest in the superficial lingual part of the alar process of the premaxillary as the palatal shelf of the premaxillary is absent in horned frogs [2, 25]

Although the lower jaw is toothless in anurans, enlarged upwardly directed fangs or odontoids are found in some neobatrachians, including the Ceratophryidae [25]. Ceratophryid odontoids are robust and fully ossified, and flank each side of the mandibular symphysis (Fig. 10). In late stage larvae, fang germs, formed by dermal bone, are differentiated on both sides of the medial process of the infrarostral cartilage before the appearance of the lower jaw bones. During earlier metamorphic stages, the fangs fuse to the dentaries [25]. In contrast to other anurans, where the odontoids constitute a laminar projection of the dentaries, in ceratophryids the fangs are ectopic ossifications integrated with the lower jaw bones having a distinctive developmental trajectory (i.e., autapomorphy, heterotypy, morphological novelty). Consistent with the unusually early hyperossification of components of the lower jaw, the postmetamorphic horned frogs appear to lack the separate and distinct mentomeckelian elements of most anurans that can rotate when the jaw is open to assist in tongue protrusion and retraction [79].

One of the most remarkable features in horned frogs is the caudal placement of the articulation of the lower jaw up to or beyond the craniovertebral joint (i.e., heterotopy and heterometry). In *Lepidobatrachus*, the jaw articulation is far behind the craniovertebral joint [7, 50]. This provides them with an enormous gape. Indeed, ceratophryids, and in particular *Ceratophrys* and *Lepidobatrachus*, have about the widest mouth openings known in extant anurans.

**Fig. 11:**
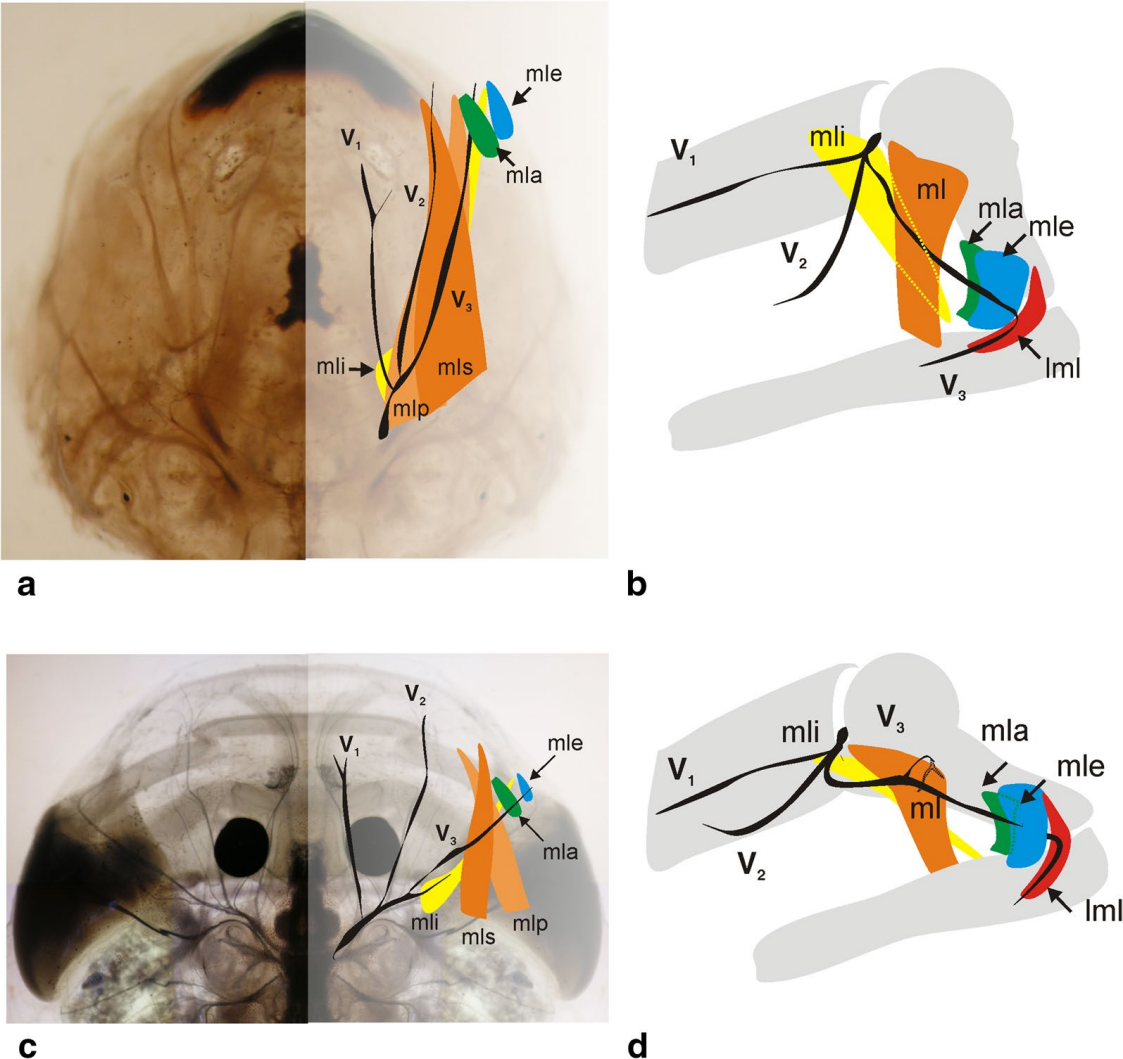
Heterotopic variation in mandibular muscles in the extant Anura, plus *Lepidobatrachus.* **a**, **b** The homology of amphibians jaw musculature was hypothesized based on muscle origin and insertions, orientation of fibers and relative position of trigeminal divisions [80]. This interpretation is applicable to both larval and adults since relations of the nerve divisions to the muscles are maintained through metamorphosis. The schema represents this condition for anurans as observed in larvae (**a** dorsal view) and adults (**b** lateral view) of *Ceratophrys cranwelli*. **c**, **d** In larvae (**c** dorsal view) and adults (**d** lateral view) of *Lepidobatrachus* spp., the trigeminal divisions (*V*_1_, *V*_2_ and *V*_3_) are positioned anteriorly to the muscles levator mandibulae, which differ from all other anurans and correlated with the posterolateral displacement of jaw suspension [7, 50]. *V*_1_ ramus ophthalmicus of trigeminus, *V*_2_ ramus maxillaris of trigeminus, *V*_3_ ramus mandibularis of trigeminus, *mla* muscle levatorae mandibulae anterior, *mle* muscle levatorae mandibulae externus, *mli* muscle levatorae mandibulae internus, *mll* muscle levatorae mandibulae lateralis, *ml* muscle levatorae mandibulae longus, *mlp* muscle levatorae mandibulae longus profundus, *mls* muscle levatorae mandibulae longus superficialis

In *Lepidobatrachus* spp., the caudally displaced jaw suspension necessitates a shift in the position of muscles levatorae mandibulae. This, in turn, changes the muscles’ relationship with the branches of the trigeminal nerve (Fig. 11). In contrast to the arrangement seen in all other anurans, in both larvae and adult *Lepidobatrachus*, the muscles levatorae mandibulae are located behind the branches of the trigeminal nerve. This shift in the placement of the muscles and their nerves has been ascribed to heterotopy [50] (Fig. 11).

**Fig. 12:**
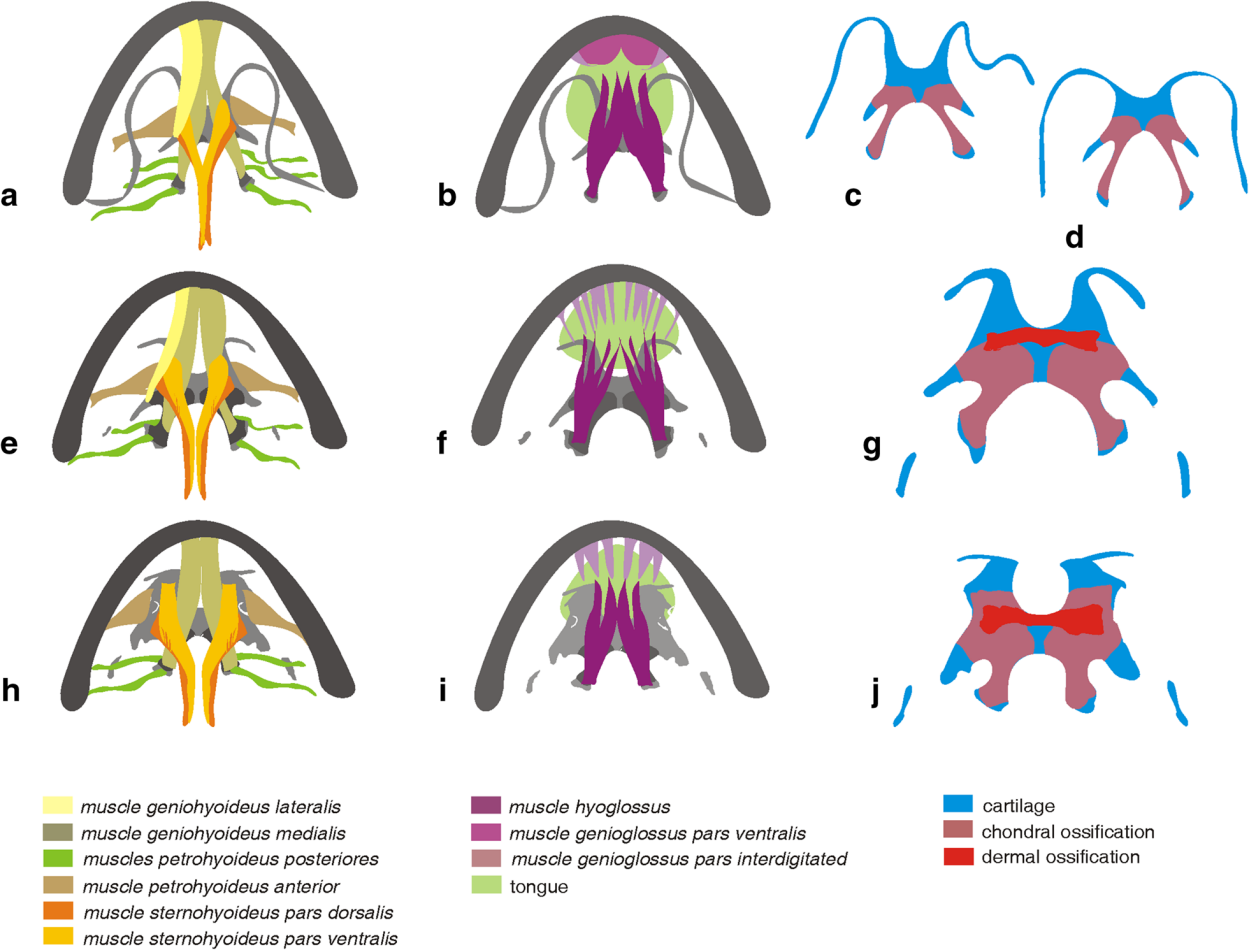
Simplified representation of the variation in the hyoid skeleton and hyoglossal muscles in Ceratophryidae. **a–d** The hyoid skeleton lacks anterolateral processes and the ossification of posteromedial processes invades the hyoid plate. **a** *Chacophrys pierottii*, **b** *Ceratophrys cranwelli*. **c** *Lepidobtrachus llanensis* and **d** *L. laevis:* Species of *Lepidobatrachus* have discontinuous ceratohyalia and a dorsal dermal bone that is unique among anurans [81]. **e–h** Variation in hyoid muscles involving the geniohyoideus, petrohyoidei posteriores, and sternohyoideus [81]. **e** *C. pierottii* and *Ce. cranwelli* present the muscle geniohyoideus divided in partes medialis and lateralis, three pairs of muscles petrohyoidei posteriores and the muscle sternohyoideus with the partes dorsalis and ventralis completely separated. This pattern of musculature is similar to that of other hyloids. f. *L. llanensis.* The pars lateralis of muscle geniohyoideus has few fibers, the anterior pair of muscle petrohyoideus is absent, and partes dorsalis and ventralis of muscle sternohyoidus have shared fibers. **g** *L. laevis.* The pars lateralis of muscle geniohyoideus is absent, and the origin of the pars ventralis of the muscle sternohyoideus is displaced anteriorly. **h–j** Variation in the genioglossus and hyoglossus tongue musculature [81]. **h** *C. pierottii* and *Ce. cranwelli* show a pattern in which medial fibers of the *left* and *right* muscle hyoglossus converge to form as a single muscle that penetrates into the tongue; and the muscle genioglossus bears two components: the muscle genioglossus ventralis that forms a solid structure and the interdigitated component, which has fibers radiating caudally from their origin on the mandible. **i** *L. llanensis*. **j** *L. laevis*. In *Lepidobatrachus* spp., the medial fibers of each muscle hyoglossus remain separate and each muscle hyoglossus conserves its autonomy. The muscle genioglossus is formed only by interdigitated components that have few divisions and loose fibers. Furthermore, the tongue in *Lepidobatrachus* is smaller than in the other genera

Some features of the hyoglossal apparatus in *Lepidobatrachus* spp. can be derived from the condition found in *Chacophrys* and/or *Ceratophrys* [81]. The hyoid skeleton of ceratophryids lacks anterolateral process, whereas the ossification of the posteromedial process is extensive with respect to other anurans (i.e., heterometry) (Figs. 1b, 12). In *Lepidobatrachus* spp., the hyoid plate is short. The hyalia are interrupted with short otic and hyoid segments, and there is an additional dorsal transverse ossification that is unique among anurans with a distinctive developmental sequence (i.e., autapomorphy, heterotypy and morphological novelty) [81].

Skeletal deviations in the ceratophryid hyoid are concomitant with changes in the hyoid musculature implying reduction in the geniohyoideus, omohyoideus and petrohyoidei posteriores muscles [81] (Fig. 12). All of these features appear to be related to a global reduction in the ceratophryids of the tongue protrusion and retraction mechanism (see additional discussion below) compared with that of more generalized frogs, which feed on smaller and faster moving prey.

## Additional developmental changes indicative of autapomorphies, heterotypy and morphological novelties

The concept of morphological novelty (i.e., heterotypy and autapomorphy) refers to new anatomical features that may acquire new functions [47, 48], and two alternative pathways for the origin of such evolutionary novelties have been proposed [82]. One pathway is the emergence of a new adaptive peak that could initially coexist with a preexisting one, which implies a change in role or function for a preexisting structure. The other involves the breaking of a developmental constraint that facilitates structural and functional integration. This would lead to a distinctive, viable and potentially unique morphology. Both processes evidently have occurred in the evolution of the Ceratophyridae and can account for much of their morphological diversity.

**Fig. 13:**
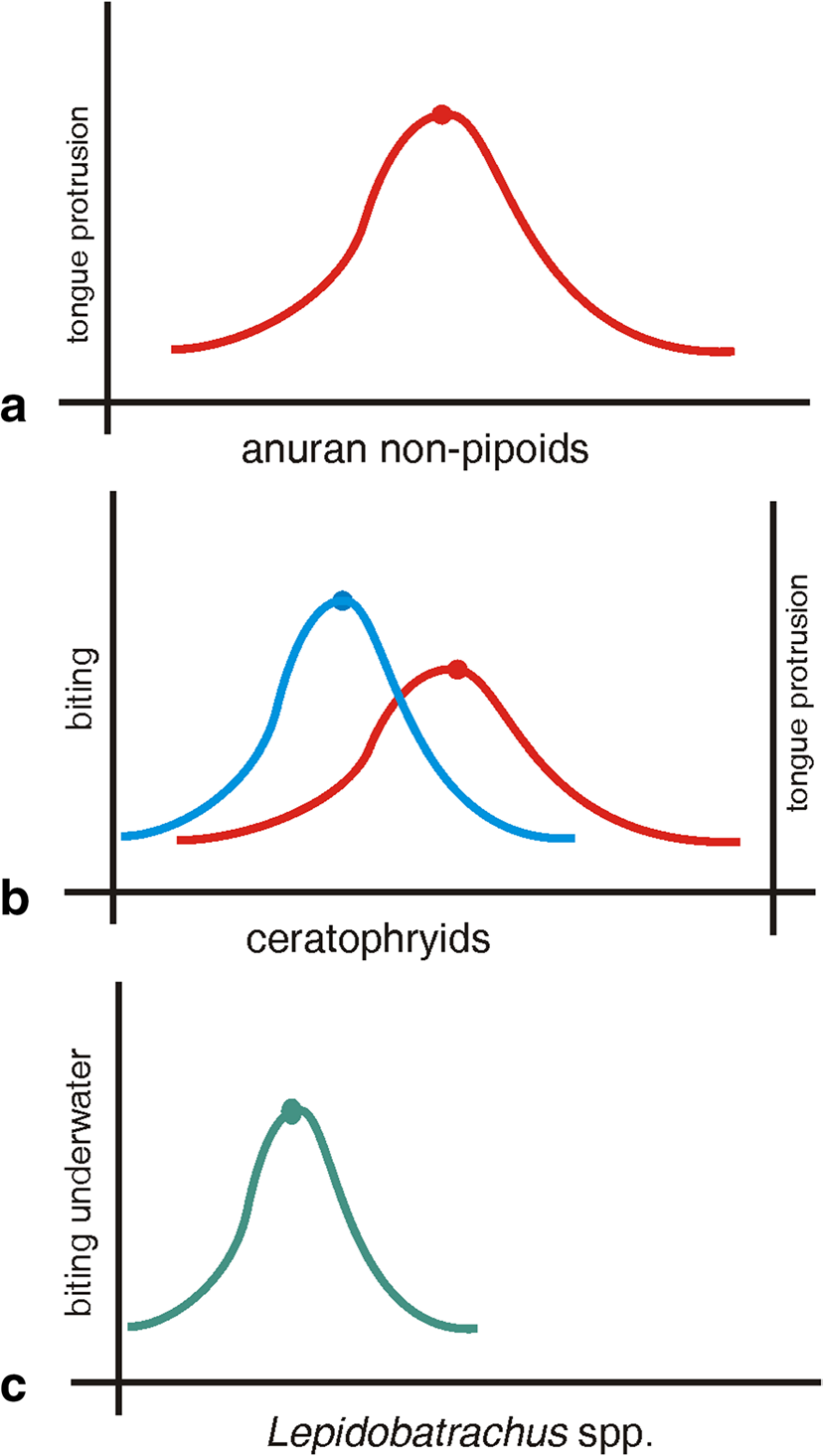
Graphic representation of the hypothesized origin and diversification of new functions in anurans. The curves represent the increment in the performance of the new function (*y* axis) through the time (*x* axis). **a** The anuran diet is composed of living prey. With the exception of the pipoids, adult anurans share a feeding mechanism in which tongue protrusion is used to capture prey. Three mechanisms of tongue protrusion have been described—mechanical pulling, inertial elongation and hydrostatic elongation—that make prey capture possible [83]. **b** In ceratophryids, the mandibular symphysis is fixed and immovable [25]. The absence of a movable joint between the mentomeckelian and dentary precludes bending of the mandibular symphysis, which is critical for tongue protrusion in most anuran taxa [79, 84]. The fangs in the ceratophryids are morphological novelties associated with these changes in the mandibular symphysis. The fangs provide the capacity for capturing and subduing large, active prey as well as serving a role in defense against predators [25, 85]. In *Ceratophrys* sp., the adhesive performance of the tongue is increased by features of its surface profile and material properties, plus mucus [86]. Thus, in horned frogs, biting and tongue protrusion act synergistically to generate the forces to catch large prey well above their own body weight (megalophagy). **c** In *Lepidobatrachus* spp., there are additional modifications from the feeding mechanism of terrestrial ceratophyrids that result from changes in development and the origin of morphological novelties, such as the dorsal dermal hyoid bone. Collectively, these changes seem to facilitate catching and swallowing large prey underwater [81]

Figure 13 depicts our interpretation of the evolutionary shift in the ceratophryid feeding mechanism away from the primacy of the tongue in prey capture, as seen in more generalized anurans. This involved the origin of morphological novelties and developmental modifications in ceratophryids for the capture of large prey. The fangs on the lower jaw, for example, appear to have evolved specifically to capture and subdue exceptionally large and active prey [25, 83]. They are integrated with other morphological traits to perform this new function. This includes the absence of pars palatina in the premaxillary, which allows the fangs to be contained within the inner face of the premaxillaries when the mouth is closed. It also includes the development of an immobile mandibular symphysis and reduction in the number of fibers in muscles associated with the floor of the mouth and tongue protrusion mechanism (e.g., muscle submentalis, muscle intermandibularis, and muscle interhyoideus)—this reduction following the sequence *Chacophrys*, *Ceratophrys* and *Lepidobatrachus* [81]. The upper jaw bears numerous spur-like and firmly anchored teeth for constraining resistant prey [75]. Lastly, the caudal displacement of the jaw suspension has led to the most distinctive feature of the Ceratophyridae namely their enormous gape [7]. Collectively, all these features in the horned frogs increase their ability, compared with that of non-ceratophryid hyloid frogs, to capture extremely large and active prey (i.e., megalophagy).

The evolutionary shift in the Ceratophryidae toward feeding on such large prey may, in part, account for their high growth rates. The most extreme shift in form and function is seen in *Lepidobatrachus.* The genus has a number of unique features in the hyoid skeleton, such as discontinuous ceratohyalia and a dermal bone attached to the dorsal face of the cartilaginous corpus of the hyoid that has not been described in other anurans (Fig. 12). There is as well a reduced number of fibers in the buccal floor muscles, and muscles that attach to the hyoid are similarly reduced in *L. llanensis* and lost in *L. laevis* (Fig. 12).

Reduction in the tongue increases room on the oral cavity to contain large prey. It is also true that, given the density and viscosity of water, prey capture with a projectile tongue is relatively inefficient. It appears that *Lepidobatrachus* has evolved a small tongue with simplified musculature as part of distinctive functional complex for aquatic suction feeding [81]. This represents a new adaptive peak (Fig. 13). Notably the unique features related to feeding in *Lepidobatrachus* are similar in both the larvae and adults; both life stages are exceptional compared with other tadpoles and adults in their ability to subdue and ingest very large, active aquatic prey.

Among ceratophryids, the increased developmental and growth rates affect all major organ systems of their larvae. Arguably, the most remarkable morphological novelties are seen in the visceral arches (e.g., the lower jaw, hyoid and brachial arches), which are essential for feeding in anuran larvae. Many of the derived features of ceratophryid larvae carryover past metamorphosis to the adults and are thus central to the overall morphological evolution of Ceratophryidae.

Anuran metamorphosis is a constrained ontogenetic period regulated mainly by thyroid hormones (THs). Each tissue responds in a selective manner to TH, with varying degrees of sensitivity to the hormones, but in general metamorphic changes are coordinated and fast [87, 88]. Several studies have shown that TH have multiple effects on organisms and evolutionary changes may occur through physiological changes in tissue sensitivity to TH, which are manifest as heterochronic changes during development [89, 90]. Thyroid glands may themselves evolve. The thyroid glands of ceratophryid larvae show signs of low glandular activity without a manifest peak at metamorphic climax as is characteristic of anurans in general [91]. In addition, different sources of TH or TH precursors from the tadpoles’ diet may influence their developmental and growth rates [91]. Many of the heterochronic changes seen in ceratophyrids appear to be due to shifts in both the concentration of TH and TH tissue sensitivity.

**Fig. 14:**
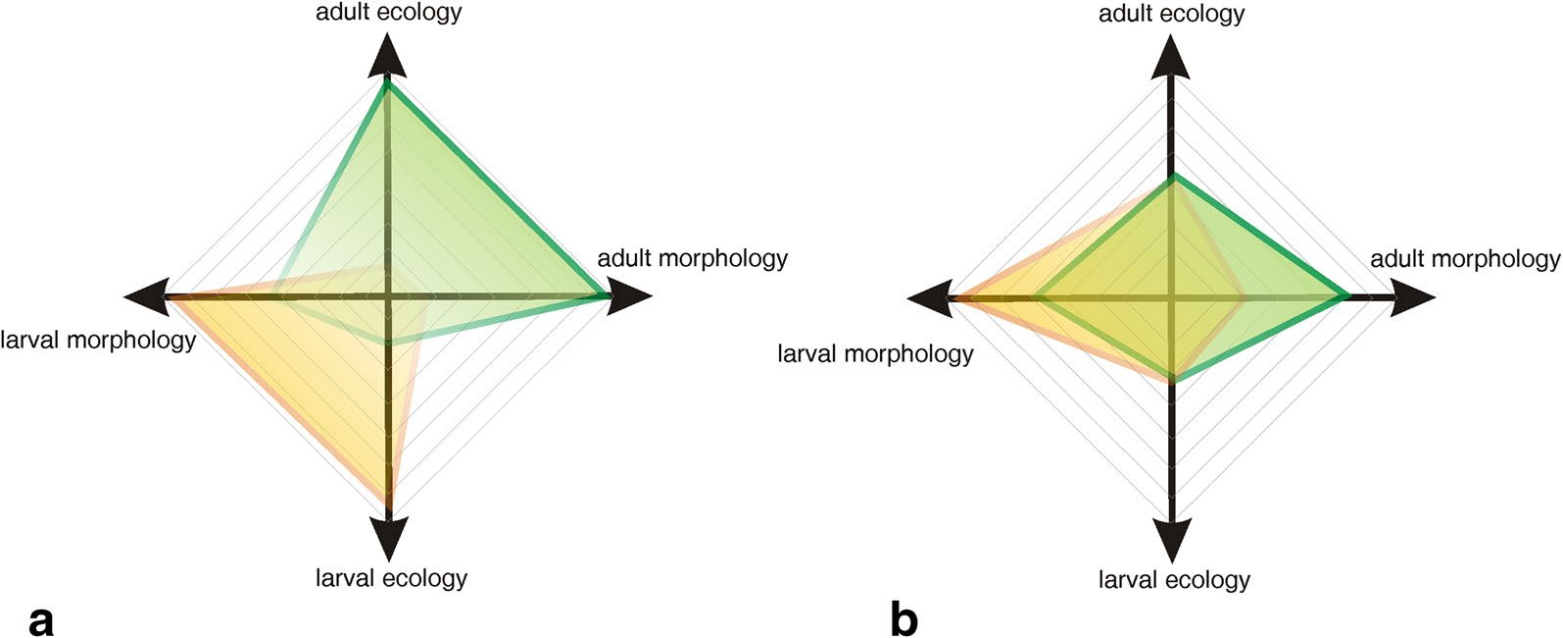
Two-dimensional graphs representing the morphology (*x* axis) and ecology (*y* axis) of biphasic anuran ontogenies. **a** Anuran larval morphology and ecology occupy the negative quadrant and are indicated by the *orange* polygon. Adult morphology and ecology are in the positive quadrant, represented by the *green* polygon. Both polygons are overlapped by metamorphosis in which there are morphological transformations in the major organ systems affecting breathing, feeding, locomotion and other behaviors, as is observed in *Chacophrys pierottii* and *Ceratophrys* spp. Metamorphosis is represented by the region around where the two axes cross. Because of the profound difference between the ecology of most larvae and adults, anurans in the middle of metamorphosis are neither as efficient in locomotion nor feeding as either the larvae or adult life form. Since anurans in transition are typically ineffective in nutritional capture and predator escape, nature selection has acted to shorten the dangerous transformational period of metamorphic climax. This is represented by the relatively small area covered by the polygons where the two axes cross in the figure. **b** The graph for *Lepidobatrachus* spp. illustrates the relatively minor ecomorphological differences between larvae and adults compared with most anurans with a biphasic lifestyle (as shown in **a**). The fast developmental rate and the precocious metamorphic morphologies in *Lepidobatrachus* tadpoles define a peramorphic larval body plan, suggesting that the free-feeding stage in *Lepidobatrachus* spp. is equivalent to metamorphic larval stages (between forelimb emergence and complete tail loss) of most anurans [11]. Furthermore, some larval features are conserved during the whole ontogeny (e.g., lateral line system) with adult stages also resembling advanced metamorphic morphologies. Because of the similarity in the life style of the *Lepidobatrachu*s larvae and adult, the typically precarious metamorphic period can be protracted; i.e., this is represented in the figure by not just the greater overlap in adult and larval polygons, but the convergence of those polygons around where the two axes cross, i.e., at metamorphosis

Figure 14 summarizes our interpretation of the origin of evolutionary novelties in *Lepidobatrachus*’s ontogeny in which shifts in metamorphosis have produced a dramatic and unique larval ecomorphology. The changes in development for the *Lepidobatrachus* tadpole have, in turn, influenced the adult body plan via a breaking of metamorphic constraints. The final result has been the origin of morphological novelties and the rise of a new adaptive peak.

Anuran larvae have historically been classified into four morphological types reflecting intraordinal macroevolution [26, 92]. Other authors [36, 37], however, have argued that the *Lepidobatrachus* tadpole is unique enough to justify labeling it as a separate morphological type. Commonly in anurans, when there has been an evolutionary departure from the classic four intraordinal types, it is by the suppression of the larval stage resulting in anurans with direct development. The ceratophryids represent, in contrast, a case where developmental variation has favored a different departure from larval constraints. This has resulted in *Lepidobatrachus* having megalophagous tadpoles unlike the larvae of any other anuran genera. The *Lepidobatrachus* body plan and life style is thus built upon morphological novelties unique among the Anura.

Despite the fact that the extant ceratophryids share numerous synapomorphies, and abundant molecular data have supported their phylogeny, they remain a monophyletic taxon with controversial in- and out-group relationships. In part, this reflects the fact that there are new structures in the Ceratophryidae that have no homology in their ancestors (i.e., autapomorphies, heterotypies or morphological novelties).

## Conclusion

The ceratophryid frogs represent an excellent model to elucidate phenotypic variation through ontogeny, and witness the many ways that heterochrony, and the breaking of developmental constraints, can yield ecomorphological novelties. The influence of this ontogenetic variation is most pronounced in the genus *Lepidobatrachus*. Indeed, because of its large size and rapid development, *Lepidobatrachus laevis* has recently been proposed as a model species in experimental studies undertaken to address a wealth of classic questions in amphibian embryogenesis [93]. Furthermore, because of its sympatry with several other ceratophyrid species (in the Gran Chaco of South America) and its well established phylogenetic relationship to those species [10], *Lepidobatrachus* stands out, not only as model species for studying developmental processes *per se*, but exceptional for studying the very evolution of those processes.

## Appendix

Glossary of major terms used in the literature to discuss the evolution and development of morphological structures in vertebrates.

The numbers in brackets refer to the key papers cited for definitions of these terms.

*Adaptation* Any feature that promoting fitness and was built by selection for its current role [40].

*Apomorphy* A character hypothesized to be uniquely derived for a particular monophyletic group [38].

*Autapomorphy* Any character hypothesized to be uniquely derived for a particular monophyletic terminal taxon [38].

*Developmental constraint* The difficulty or impossibility of producing certain variations from a given starting point [39].

*Developmental drive* The ease of producing variation in development in particular directions, opposite and complementary, to developmental constraint [39].

*Evolution* Unidirectional (non-cyclic) organic change through multiple generations [38].

*Evolutionary novelty* New structure, new function, new adaptive niche [47–49].

*Exaptation* Any feature that now enhances fitness but was not built by natural selection for their current role [40].

*Growth heterochrony* The occurrence of developmental changes in the relationship of size [42–44].

*Heterochrony (other timing)* Change in the developmental timing for a part of an organism with regard to the time for development of that part in the ancestral ontogeny [39].

*Heterometry (other amount)* Change in the amount (in size or proportion) of some part of an organism with regard to its proportions in the ancestral ontogeny [39].

*Heterotopy (other place)* Change in the anatomical location of a structure in an organism with regard to the same part in the ancestral ontogeny [39].

*Heterotypy* The absence of any shift in the time of appearance, location or proportions of a structure in a current organism compared with the ancestral ontogeny [39].

*Hypomorphosis/hypermorphosis* Evolutionary processes producing interspecific changes in growth rates with consequences in adult size [46].

*Homology* Structures having the same origin, position, shape or composition in two or more organisms [38].

*Homoplasy* Incongruent data which, on grounds of parsimony, cannot be explained as homology (i.e., as due to common ancestry) [38].

*Morphological change* Any variation between the same kind of building blocks (i.e., organs, tissues or cells), among individuals or between species [41].

*Morphological novelty* A new structural element in a body plan that has no homology in the ancestor or in the same organism (serial homology) [47, 48].

*Natural selection* Differential survival of the variants across generations [38].

*Ontogeny* Developmental trajectory (series of forms) of an organism from its starting point to maturation [39].

*Paedomorphosis/peramorphosis* Evolutionary processes producing interspecific changes in developmental rates with consequence in the adult shape [45].

*Parsimony* Methodological tool to select the preferred hypothesis based on the fewest assumptions about a data set [38].

*Phylogeny* A pattern of evolutionary relationship between three or more taxa [38, 39].

*Sequence heterochrony* As the developmental trajectory is conceptualized as a series of discrete events, sequence of heterochrony is demonstrated when the temporal position of an event changes relative to other events in that sequence [42–44].

*Synapomorphy* A trait shared by all members of a monophyletic group set [38].

## Abbreviations

dpha: distal prehallical element
ep: epidermis
ho: hypodermis
ml: muscle levatorae mandibulae longus
mla: muscle levatorae mandibulae anterior
mle: muscle levatorae mandibulae externus
mli: muscle levatorae mandibulae internus
mll: muscle levatorae mandibulae lateralis
mlp: muscle levatorae mandibulae longus profundus
mls: muscle levatorae mandibulae longus superficialis
mm: millimeter
mx: maxillary
mxt: maxillary teeth
pmx: premaxillary
pmxt: premaxillary teeth
pph: proximal prehallical element
sc: stratum compactum
ss: stratum spongiosum
TH: thyroid hormones
*V*_1_: ramus ophthalmicus of trigeminus
*V*_2_: ramus maxillaris of trigeminus
*V*_3_: ramus mandibularis of trigeminus
